# Producing concept-motivated signs supports learning of STEM in American Sign Language

**DOI:** 10.1038/s41539-026-00418-6

**Published:** 2026-04-11

**Authors:** Rachel Sortino, Christina Kim, Thalia Guettler, Katie McClyman, Lorna Quandt, Alicia Wooten, Rachel Pizzie

**Affiliations:** Gallaudet University, Washington, DC USA

**Keywords:** Education, Language and linguistics, Language and linguistics, Psychology, Psychology

## Abstract

Deaf and hard of hearing students often lag behind their hearing peers in STEM classes, in part because of a lack of STEM learning resources available in sign language. Past research shows the benefits of embodied cognition through iconic gestures for hearing students. We investigated whether signed lessons that emphasized connections to English or to concepts supported embodied learning of STEM topics. In Study 1, we developed and validated pairs of lessons in two signing styles: English-motivated (EM) and concept-motivated (CM). In Study 2, we compared learning from those two signing styles. Participants’ scores increased from pre- to post-test, indicating learning, but there were no differences based on signing style. However, when we examined participants’ signed summaries, we found that increased production of CM signs, but not EM signs, was related to higher post-test scores. This result suggests that the benefits from embodied learning emerge when learners produce the concept-motivated signs themselves.

## Introduction

In the United States, there is an increasing need for students to develop expertise in science, technology, engineering and mathematics (STEM) to thrive in a world that requires expertise in technological and analytical thinking skills. However, US students are not meeting benchmarks for performance in STEM, at least as measured by standardized test scores. This indicates that the education system may not be adequately setting up students for success in STEM fields. According to an analysis of recent National Assessment of Educational Progress (NAEP) scores, only 30% of non-disabled students scored at “proficient” or above in mathematics^1^, 38% scored at or above “proficient” in science^2^, and 51% scored at or above “proficient” in technology and engineering literacy^3^. However, the situation is even worse for students with disabilities. For grade 8 students with a disability, only 7% scored at or above “proficient” in math^1^, 12% in science^2^, and 14% in technology and engineering^3^. These scores indicate that a vast majority of disabled students in the US are performing below “proficient” standards for STEM skills and knowledge. As such, they would likely benefit from changes to STEM pedagogy designed to improve their learning and conceptual understanding.

Nationwide data, like that collected through the NAEP, provides information on students who experience a wide variety of disabilities. Smaller-scale studies have shown that deaf and hard of hearing (DHH) students, on average, perform behind their hearing peers in math and science^4,5^. One strategy that has been used to support STEM learning for non-disabled students is “embodied learning.” Embodied learning focuses on how students can use their bodies to create additional mental representations of concepts they are learning. For example, using gestures creates a visuospatial and physical connection to an underlying STEM concept. Existing research with hearing students showed that using iconic gestures that resemble a concept supported STEM learning^6–9^. However, more investigation is needed to understand how iconic signs (i.e., signs that visually represent an action, object, or concept) in American Sign Language (ASL) could support the learning process in signing students. This is of particular importance because there are currently many efforts across the country to develop a more standardized STEM lexicon in ASL. These newly developed signs may use strategies like iconicity to help convey scientific information through signed language^10,11^. By utilizing the unique features of ASL to support better STEM education, our aim was to investigate ASL signing styles that incorporated two categories of STEM signs: English-motivated (EM) and concept-motivated (CM). Our aim in Study 1 was to create and validate STEM video lessons in these two styles. In Study 2, we compared learning between both types of signing styles in order to analyze how EM and CM signing styles might support STEM learning.

For DHH students, research and interventions often focus on reading and writing skills and academic success^12^. However, there is a growing body of evidence that shows language ability and, in particular, age of accessible language (i.e., ASL) exposure explains delays in DHH children’s math skills^12–14^. For example, one synthesis of 23 articles about DHH students’ performance in math found that at an early age, there were no performance gaps for non-language-based tasks (i.e., tasks that did not require counting^15^). This provides support for the idea that it is not hearing loss that relates to delays for DHH students, but rather a lack of full access to language. Additionally, in a recent longitudinal study by Cawthon et al. (2023), they analyzed Measures of Annual Progress (MAP) scores for 351 DHH students, the majority of whom attended schools for the deaf, where ASL is typically used for instruction. While DHH students’ scores were, on average, lower than their hearing peers in this sample, DHH students followed a similar growth curve to their hearing peers as they progressed from second to eighth grade. These results implied that “deficit” models that assumed DHH students inherently lack math ability were incorrect. Instead, these results showed support for a “delay” model, suggesting that DHH students are not inherently less skilled at math, but rather that the high rates of delayed access to language contributed to delays in acquiring math skills. Notably, for the students in this sample, who were largely taught in ASL, the gap between hearing and deaf students did not increase over time, showing that with enough time and appropriate support, it is reasonable to assume that DHH and hearing students’ STEM performance should be equivalent^16^. Though little research has been done investigating growth trends in science, technology, or engineering skills in DHH students, deficits in these domains can likely be attributed to delayed language access.

The impacts of delayed language access on STEM performance are further amplified by the lack of resources explaining STEM concepts in ASL. DHH students rarely have access to STEM content in their native language^11^. Classroom resources (i.e., textbooks, worksheets, PowerPoints, assessments) are generally provided in English. Online resources like educational videos are often not accessible to DHH students because they lack high-quality captions. Even videos with captions require DHH students to learn through English, which is a secondary or nonpreferred language for many DHH students^17^. This lack of resources in ASL is particularly impactful on DHH students who attend “mainstream” schools with hearing peers and access class content through ASL interpreters. For more advanced STEM content, many ASL interpreters do not have the training or deep knowledge of STEM coursework to conceptually interpret the course content taught by the instructor. In these cases, interpreters will rely on transliteration, essentially using a mix of fingerspelling and non-technical translations of signs to get the English message across. This strategy often results in losing the actual concept and the natural grammar of ASL^18^.

One study investigated how this emphasis on transliteration during interpretation could impact learning by learning from direct instruction from a signing teacher compared to indirect instruction through an interpreter. Researchers had middle school students watch videos of a deaf teacher signing several lessons. This was compared to learning from a professional interpreter signing a lesson and interpreting from English into ASL. Even though the interpreter was a highly qualified, native signer, with 25 years of experience, students still showed significantly more improvement from pre-test to post-test after the direct-instruction videos^19^. While companies like Khan Academy are adding ASL versions of their online lessons^20^ and groups like ASLCore are developing STEM dictionaries in ASL^18^, we know very little about what styles of direct instruction in ASL are best for learning STEM content for DHH students.

The lack of a consistent lexicon for STEM concepts creates additional barriers for DHH students. The lack of consistent signs between teachers, grade levels, and scientific disciplines has created barriers to building more advanced conceptual understandings for signing students. For example, many DHH students are taught different signs for the same concepts over the course of their academic experiences. Imagine if, in the third grade, you learned about the phases of matter as “hard things,” “wet things,” and “air things.” Then, in fourth grade, your teacher teaches about the phases of matter as “solids,” “liquids,” and “gases.” However, your teacher never clarifies that these are the same concepts as the “hard”, “wet”, and “air” concepts that you had learned about the previous year. This lack of consistency creates an additional barrier for students, requiring them to independently make connections to build on prior knowledge. If students cannot make these connections between the different terms, it prevents them from utilizing existing knowledge structures to build and then elaborate knowledge. Instead, they have to start from a new place every time^21^.

While teachers of deaf students base their instruction on standards used for hearing students, which intentionally scaffold and spiral key vocabulary in English^22^, the same scaffolding and standardization are not available for ASL. The impacts of this lack of standardization can be compounded for DHH students in mainstream educational environments. Some students in these environments may have different ASL interpreters from week to week, or day to day, all of whom might use differing signs to explain the same concept. This lack of consistency is part of what has motivated a number of groups to develop ASL STEM lexicons to create shared STEM terminology that is more widely adopted and used^10,23–25^.

**Fig. 1:**
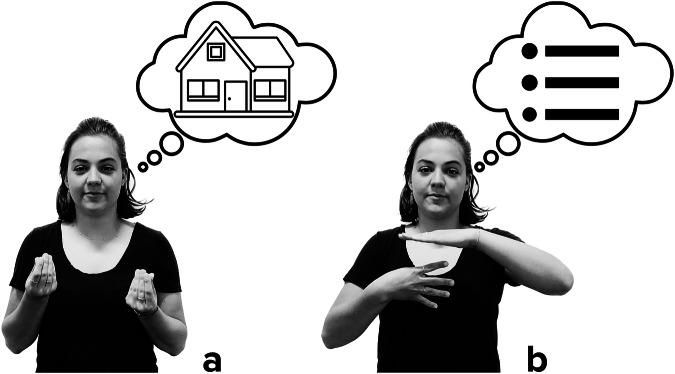
Two signs for “property”. Signs for vernacular meaning of “property” (**a**) and technical meaning of “property” (**b**).

**Fig. 2:**
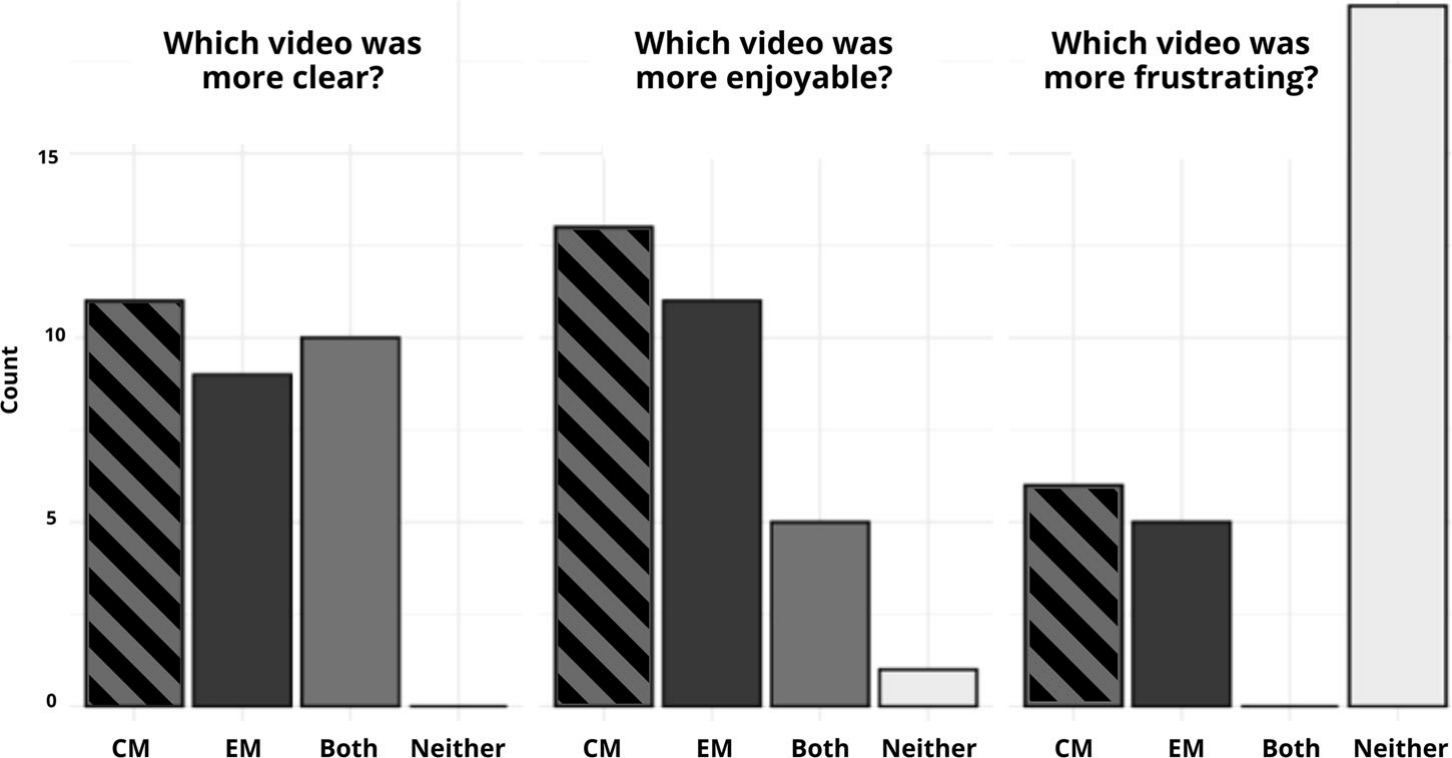
Distributions of participant responses to which style of video was clearer, more enjoyable, and more frustrating to watch. Histograms of participant responses making ratings of concept-motivated (CM) and English-motivated (EM) videos. Results across ratings of clarity and enjoyability suggest that there was not a particular signing style that was preferred compared to the other, with similar numbers of participants showing a preference for either CM or EM styles. For frustration, the majority of participants indicated that neither video was more frustrating.

Developing STEM lexicons is a complex linguistic issue^18^. A major challenge for consistency is a debate over whether signs should emphasize connections to English, or connections to more visuospatial, conceptual ideas^17^. English-motivated (EM) signs generally fall into three categories: (1) “borrowed” from signs for non-technical definitions of English words (e.g., Fig. 1a), (2) initialized versions of signs that use the first letters of the English spelling, or (3) fingerspelled using the manual alphabet. These types of signs primarily focus on making connections to the STEM concepts through English words. This approach is often driven by the fact that English is the de facto language of science, so knowing how to discuss a concept in English is the primary goal^17^.

Critics of this approach have emphasized that these signs can exacerbate the confusion caused by differing technical and non-technical definitions of English words^26^. Consider the example of “property” (Fig. 1). One common non-technical definition for “property” is a possession, such as land. As such, the non-technical ASL sign for “property” looks like someone rubbing dirt with their fingers (Fig. 1a). This sign is commonly used in STEM classrooms when talking about “properties” of matter, even though this is a conceptual mismatch. In this technical context, “properties” is defined as a list of characteristics and has no connection to the idea of land. The concept-motivated (CM) sign for “property” in this context shows a list (Fig. 1b), fitting the technical definition and avoiding conjuring the non-technical meaning for the word^18^. This difference in technical and non-technical definitions is not unique to students learning through ASL; it also occurs in English. However, the physical resemblance of the sign to the non-technical definition (i.e., iconicity) that is embedded in many of the non-technical signs could create additional opportunities for misunderstanding for DHH students when they are used in technical contexts.

By contrast, the iconicity of CM signing is more closely matched to the scientific or technical meaning of STEM signs, largely because they have been developed to use visuospatial depictions to convey content-related information. The iconicity of CM signs has the potential to support learning, similar to iconic gestures^6–8^. The benefits of iconic chemistry signs were illustrated when researchers developed CM organic chemistry signs for interpreters and DHH students to use in organic chemistry classes^24^. After implementing CM signs in the classroom, DHH students’ grades surpassed those of their hearing peers. Importantly, this was not simply a matter of students memorizing signs for specific English words and earning better grades. Because the signs were developed to make connections between concepts more visuospatially apparent, their use also modified the way interpreters and students signed about the concepts. For example, instead of fingerspelling types of reactions, interpreters now had signs that represented the reactions and made the similarities between the transition states of different reactions more explicit. The fact that the new signs made connections between concepts more visible was cited by the authors of the study as a potential reason the DHH students performed better than hearing students on course exams after the introduction of these new signs^24^. Whereas EM signs help students make connections with the English words, CM signs capitalize on the visuospatial nature of ASL to make broader conceptual connections more explicit^18^. Our research investigated whether differences in the relationship between EM and CM signs and the underlying STEM concepts could be leveraged to support embodied learning.

Embodied cognition in the classroom is focused on using physical experience with the body to make connections between abstract concepts and physical representations of those concepts^27^. In STEM classes, this is particularly important since many concepts are difficult for students to envision because they are often abstract (e.g., derivatives in calculus), invisible (e.g., forces acting on a building), or on a scale that is hard to comprehend (e.g., minuscule atoms or massive galaxies). There are various ways to use embodied learning to help address these challenges since embodiment naturally exists on a spectrum^28^. Any learning in a classroom is always embodied to some extent because students have first-person experiences, sensations, and memories while engaged in learning. However, the level of conceptual embodiment varies in terms of how many modalities are used and the extent to which students’ action is connected to their thinking and learning. For example, while having students get up and move around the classroom is an excellent strategy for improving engagement, the act of moving does not automatically support embodying a concept^29^. However, when students intentionally use their bodies to process and learn new information, they create an additional, embodied representation of the concept^30^.

Gestures produced by teachers are on the lower end of the embodied learning spectrum. Students observe but do not typically imitate these gestures with their own bodies. Nevertheless, many studies have shown that teachers can use accurate, meaningful gestures to support learning^9,31,32^. Because students are not actively using their bodies to imitate these gestures, these benefits are likely not directly from physical embodiment but may instead support learning through reduced cognitive load. Specifically, teachers’ gesturing can make it easier for students to make connections between different ideas by making the ideas and the connections more visible, spatial, and concrete^7^. Similarly, when students produce gestures on their own, it creates additional sensorimotor representations of a concept and allows students to use their bodies to supplement or even replace verbal or written communication, reducing the cognitive load of the task^33–36^. The use of gestures can also predict readiness to learn in both hearing and signing DHH students^37–39^. Specifically, when children use gestures to express what they know, sometimes these gestures express ideas that are not readily or correctly expressed via language, creating a gesture-language mismatch. When children produce gestures that do not match what they have spoken or signed, these gestures indicate a readiness to engage with the task at hand. As such, they are more likely to succeed after instruction than students who had fewer gesture-sign or gesture-speech mismatches. This finding was mirrored across spoken and signed languages, indicating that gestures are processed differently than signs, even though they are in the same manual modality.

Finally, at the high end of the embodiment spectrum is “hands on learning.” A study by Kontra et al. (2015) found that when participants physically engaged with a spinning wheel to learn a about physics, they performed better on related assessments than students who only observed the system. More significantly, their performance on the task during a functional magnetic resonance imaging (fMRI) scan correlated with motor cortex activation while taking the assessment^40^. This correlation indicates that their improved performance was specifically linked to retrieving memories about their physically embodied learning experience.

While the research thus far on the science of learning clearly shows that embodied experiences can support STEM concept development, we do not yet know where CM and EM signing fall on the embodied learning spectrum. EM signs could support more embodied representations that connect to the “language of science,” English, boosting familiarity with terms^17^. Because most of the materials students use to learn about STEM concepts (e.g., videos, books) are in English, making connections directly to English could maximize their ability to relate what students read and what they sign^17^. Additionally, most measures of STEM understanding are tested using English, so being able to connect their embodied experience to English could lead to more success on assessments. Alternatively, CM signs are able to make the concept clearer through visuospatial depiction, making the connections between concepts more transparent. For example, the CM signs for the phases of matter use patterns of motion and handshapes that communicate how the phases and their transitions are related (see supplementary information for videos). Combined, the conceptual and visuospatial advantages afforded by CM signs could provide needed support to build a foundational understanding of STEM concepts^18^. Additionally, when students see and use either CM or EM signs, they could create an additional connection to another sensory modality within their mental representation of the concept^27,30,41^. For EM signs, their actions could create a stronger connection to the concept through English words. While for CM signs, their actions could create a stronger connection to the concept directly. In the present research, we investigated whether the indirect connection to the concepts through English or a direct connection to concepts was more critical for supporting understanding of STEM information.

Across two studies, our goal was to explore how differences in signing styles related to the learning of STEM content. For Study 1, we created and validated video lessons for various STEM topics in two distinct ASL signing styles that we call English-motivated (EM) and concept-motivated (CM). Both signing styles use fluent ASL in distinct ways to explain the STEM concepts. After validating the videos through reiterative feedback from experts and key stakeholders, in Study 2, we measured if participants were able to learn from these video lessons. We compared whether participants in an in-lab study were able to increase their learning after watching the videos. Using a within-participant design, we measured their knowledge when learning from both CM and EM lessons. Overall, our goal was to explore whether these signed video lessons could lead to learning on a short timescale, given that the video lessons were all four to six minutes in length. Moreover, we wanted to explore how the CM and EM signing styles led to any differences in learning, including how much participants were able to incorporate the signs into their knowledge about the topic.

## Results

### Study 1

Study 1 focused on developing and validating ASL training videos for four different STEM topics in two different signing styles (EM and CM). As addressed above, there are a multitude of STEM educational videos available in English and even some with ASL interpretation. However, there are very few resources for students that provide direct instruction in an accessible signed language like ASL. As such, we wanted to focus on developing lessons using direct ASL instruction as opposed to an ASL interpretation of existing spoken English videos. We chose to make fully separate translations for each video topic as opposed to simply swapping out the key CM and EM signs to create more coherent, standalone lessons in ASL for each topic. As such, the CM lessons used a more three-dimensional, visuospatial depiction of scientific content in ASL such as showing that a solid is molecules packed together, limiting molecular movement. The EM lessons used a more linear ASL signing style and more fingerspelled terms and literal English translations (i.e., non-technical, vernacular sign for “property,” Fig. 1a). We hypothesized that if both styles of signing provided adequate and clear explanations of the content, there would be no clear preferred style when participants reviewed them for clarity, enjoyment, and frustration.

In order to test if differences in signing style influence the learning of STEM content, we developed training videos in different CM and EM styles of signing. Our goal for these training videos was to create two versions for each lecture that were equally clear but linguistically distinct explanations of the content. The visual aids and English “scripts” used to develop the content for each pair of videos were identical, but the CM and EM strategies for language use in ASL presented the content in two different ways. We surveyed teachers of the D/HH, ASL teachers, ASL interpreters, and ASL linguists (*N* = 30) to judge the relative clarity and enjoyability of different pairs of videos. Each reviewer was assigned to one of the four topics and viewed both the EM and CM videos and answered a series of multiple choice and open-ended questions comparing them.

Because of the relatively small number of reviewers per topic area (*n* = 7–9 per video pair), we analyzed the preferences for EM and CM styles for all four topics combined instead of by individual topic. Prior to combining these data, we explored the data for each topic. For each topic, at least two reviewers expressed preference for either CM or EM style of video, so there were no topics where all reviewers preferred one version over the other, bar graphs showing this breakdown can be found in Supplementary Fig. 1. In order to analyze our data, Chi-Square tests were used for non-parametric response data, testing whether the distributions of responses differed from a hypothesized distribution across the four response options (video A, video B, both, neither). We compared the distribution of our response data to the following models: (1) an equal chance distribution of responses across the range of four responses and (2) equally distributed responses across the range of 3 affirmative responses (video a, video b, both) and minimal response to the negative option (neither). A bar graph showing the summarized reviewer responses is in Fig. 2.

### Clarity

Participants were asked to judge which video in their assigned pair was most clear. Across all video pairs, a slight majority preferred the CM versions (*n* = 11), while a similar number of reviewers preferred the EM version (*n* = 9), and the remaining reviewers rated them as being equally clear (*n* = 10). None of the reviewers reported that neither video was clear. Observed frequencies are significantly different than the null hypothesis (all responses will be equal, *X*^2^(3) = 10.27, *p* = 0.016). We then compared our results with a predicted model of an equal distribution for preferences for “CM,” “EM,” and “both” with a limited number of responses for “neither” and found no significant difference between our data and this hypothesized model (*X*^2^(3) = 0.51, *p* = 0.918). This result indicates participants judged the different signing styles to be approximately the same level of clarity.

### Enjoyability

Participants were asked to judge which video in their assigned pair was more enjoyable to watch. Similar to the question for clarity, a slight majority of reviewers preferred the CM videos (*n* = 13) while slightly fewer enjoyed the EM version more (*n* = 11). Five participants found both signing styles equally enjoyable, while one participant found neither video enjoyable. Observed response frequencies were significantly different than the null hypothesis (all responses will be equal, *X*^2^(3) = 12.13, *p* = 0.007). We then compared our results with a predicted model of an equal distribution for “CM,” “EM,” and “both” with a limited number of responses for “neither” and found no significant difference between our response data and the hypothesized model (*X*^2^(3) = 5.15, *p* = 0.161). This result indicates participants reported a similar level of enjoyability for each signing style.

### Frustration

Participants were asked which video in their assigned pair was more frustrating to watch. A majority of participants (*n* = 19) reported that neither video was frustrating to watch. Of the remaining reviewers, six found the CM version more frustrating, while five found the EM version more frustrating. None of the reviewers found both signing styles frustrating. Observed frequencies are significantly different than the null hypothesis (all responses will be equal, *X*^2^(3) = 26.27, *p* < 0.001). We also compared our results with a predicted model of an equal distribution for “CM,” “EM,” and “neither” with a limited number of responses for “both,” and found a significant difference (X^2^(3) = 12.63, *p* = 0.006), such that our distribution data were significantly different from the proposed model. Finally, we compared our results with a model predicting half of the responses to be for “neither” with an equal distribution of remaining responses split between “CM” and “EM.” This comparison showed no significant difference between the predicted model and our data (*X*^2^(3) = 2.54, *p* = 0.468). This result indicates that most participants did not find either signing style frustrating, and those that found one style or the other frustrating were equally balanced across CM and EM.

### Descriptions of signing styles

In addition to asking reviewers to choose which videos they found more clear, enjoyable, and frustrating, we also asked them to describe the differences they noticed between the videos using a written text response. In order to analyze the themes in these text responses, the research team sought an objective language analysis that might be less influenced by subjective demand characteristics of the research team. We submitted deidentified text responses through a large language model^42^ to identify themes, which were reviewed for accuracy by a member of the research team^43^. The themes summarized across all four topic videos in the EM style included multiple comments about the clarity of the singing style, that they followed an English sentence structure, and that they used more fingerspelling. Themes for the CM videos include comments about the use of conceptual vocabulary signs, the use of space and classifiers, and the fact that they were overall more visual. Similar to the multiple-choice comments, each signing style had some reviewers who expressed a preference for one over the other.

### Interim discussion

Our goal was to create four novel STEM lessons in two distinct ASL signing styles for a total of eight video lessons. After verifying the accuracy of the content and appropriateness of the signing styles with our research team and external content experts, we ran an online validation study with deaf education experts (deaf education teachers and ASL teachers, linguists, and interpreters). The results from that study indicated that we succeeded in creating video pairs that provided equally accurate, clear, and appropriate explanations of various STEM topics. Participants rated the EM and CM versions of our video lessons as equally clear, enjoyable, and frustrating, with the majority of participants noting that neither video was frustrating to watch. This validation analysis indicated that these videos would be appropriate for use in Study 2 without concern for any differences in learning being attributable to a baseline difference in quality. Additionally, reviewers’ descriptions of the differences in the signing styles matched our goals for distinguishing between CM and EM even though participants were naïve to intentional differences in video design.

### Study 2

**Fig. 3:**
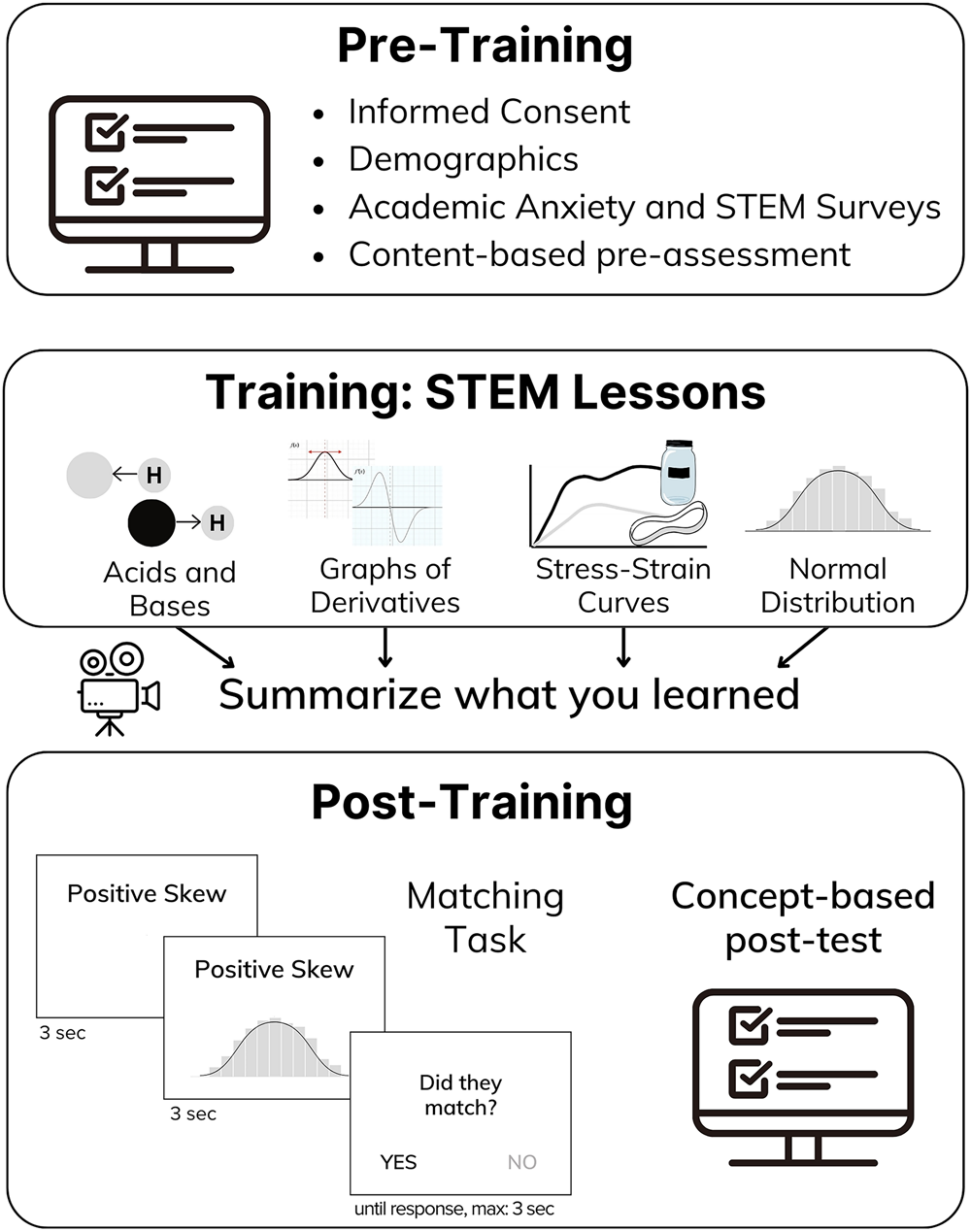
Graphical overview of Study 2 procedures. Overview of study procedures showing pre-training, training, and post-training.

In Study 2, our aim was to understand how utilizing visuospatial language might augment learning and understanding of STEM topics. The primary goal for Study 2 was to use videos developed during Study 1 to investigate if there were differences in learning based on the signing style (EM versus CM) of the video lessons. Participants (*N* = 30) completed a within-participant experimental study that measured learning across STEM topics with a pre-test, a learning phase with the ASL video lessons, and post-test comparison (see Fig. 3 for a graphical overview of the procedures). As another test of learning, participants also completed a word-picture matching task that tested concepts learned during the video lessons. Each participant completed a pre-test to assess baseline knowledge on all four STEM topics. During the training phase, participants viewed ASL video lessons for two topics in the CM signing style and two topics in the EM signing style. After each video lesson, participants signed summaries of what they learned after each lesson, and these signed summaries were video-recorded and analyzed. After the learning phase, participants completed a matching task, which presented paired pictures and English terms from the video modules. Finally, participants completed a post-test, which used a similar structure to the pre-test to compare scores and the change induced by learning from the video lessons. We compared pre- and post-test performance generally and across our two signing styles. Our hypotheses were as follows:

- Participants would show improvement from the pre-test to the post-test, indicating a change based on learning from the video lessons.
- Participant performance on the matching task would be correlated with post-test performance, but not pre-test performance, indicating that both the matching task and post-test scores would be reflective of learned knowledge gained by studying the video lessons.
- Participant improvement from pre-test to post-test would differ for the two signing styles. We predicted that knowledge learned in CM video lessons would be associated with more score improvement, indicating improved learning.

### Linear mixed-effect model development

To measure learning, we used a linear mixed-effects model (LMM). All statistical analyses were completed in R^44^ via the lme4 package^45^. These models make it possible to measure the fixed effects of our experimental conditions (i.e., signing style) while accounting for random effects (i.e., participant individual differences, differences in learning across the four topics). Signing styles were counterbalanced across topics and between participants. Thus, when we summarized our data, CM and EM scores include different topic areas between participants (see Fig. 7). This counterbalancing allowed us to compare the effects of CM and EM signing styles regardless of the content, because the effects of the signing style cannot be fully attributed to differences created by the topic of the lesson. For example, some participants’ CM scores include “acids and bases” and “the normal distribution,” and other participants’ CM scores include “stress and strain curves” and “graphing first derivatives.”

Our primary analyses explored pre- and post-test scores as a primary outcome as a measure of learning. First, we used an LMM predicting test scores on the pre- and post-tests with the topic (acid/base, normal distribution, graphing derivatives, stress and strain curves) as a fixed effect and individual differences (ID) as a random effect: *score ∼ topic* + *(1∣ID)*. With this model, we found that the different topic areas predicted significant differences between scores (*X*^2^(3) = 12.94, *p* = 0.005). Thus, we added “topic” as a random effect to our additional models (described below) to include variability attributed to learning across the variety of STEM topics.

Our analyses also explored performance on the matching task as a measure of learning. Like our LMM for pre- and post-test scores, we computed an LMM predicting accuracy on the matching task as the outcome measure. We also included response time (RT) as a fixed effect and individual differences as a random effect: *accuracy* ~ *RT* + *(1* | *ID)*. We found that response time significantly predicted accuracy on the matching task (*X*^2^(1) = 36.07, *p* < 0.001). We kept response time as a fixed factor of non-interest in the model since the relationship between accuracy and response time is non-random (i.e. people generally respond more quickly when they are confident). We then tested topic area as a fixed effect in the model: *accuracy ~ topic* + *RT* + *(1* | *ID)*. Again, we found that different topics significantly predicted differences in accuracy on the matching task (*X*^2^(3) = 37.59, *p* < 0.001). So, we included “topic” as a random effect in our additional LMMs (described below) to account for this variability across topic areas.

Taking these preliminary analyses into account, we developed two base LMMs that we built on throughout these analyses. To measure learning using the pre- and post-tests scores as an outcome measure, our base model included random effects attributed to topic and individual differences for each participant: *score* ~ *(1|topic)* + *(1∣ID)*. For the matching task, our base model included accuracy as the outcome measure, RT as a fixed factor, and random effects attributed to topic and individual differences attributed to each participant*: accuracy* ~ *RT* + *(1|topic)* + *(1∣ID)*.

### Measuring learning

**Fig. 4:**
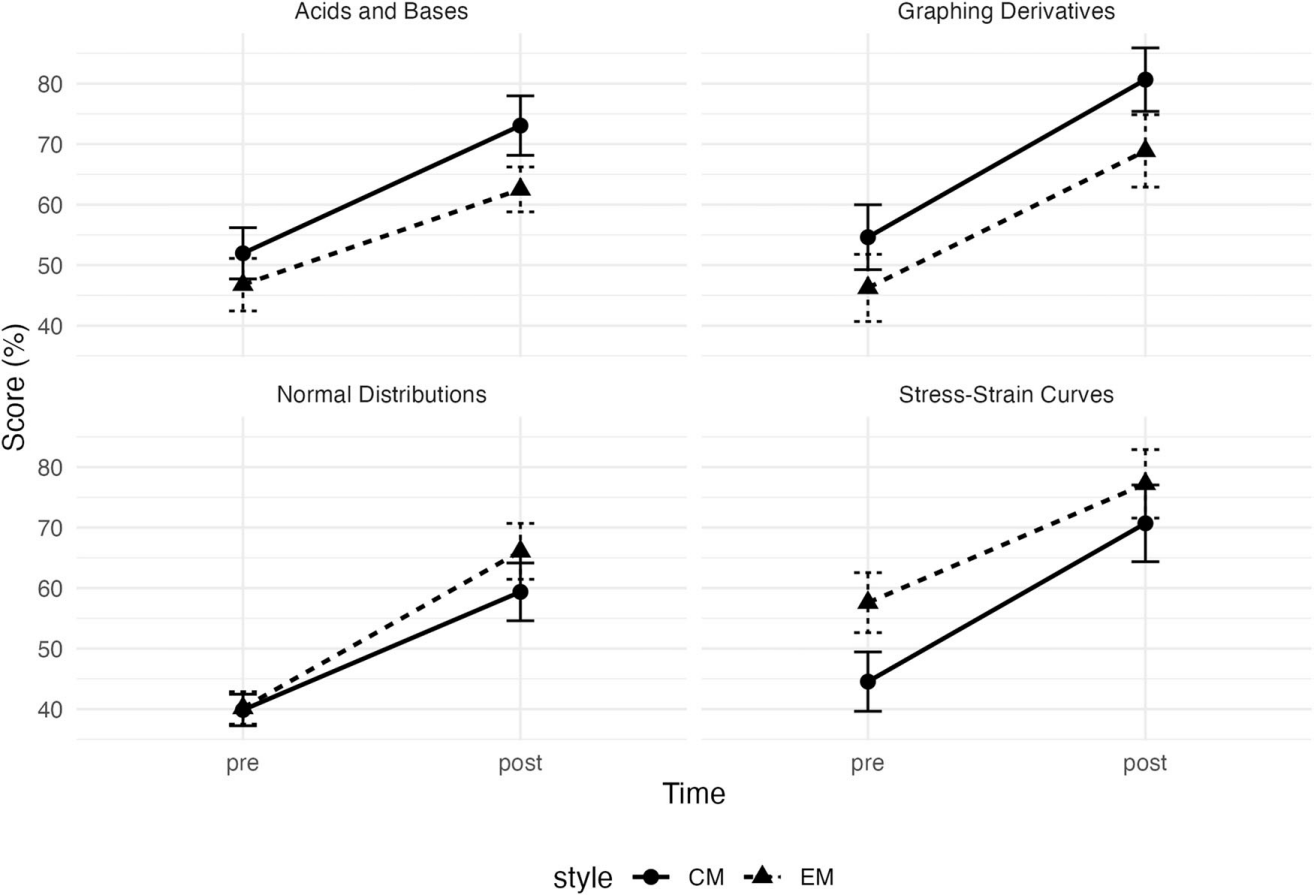
Average pre- and post- test score on content assessments across all content areas and signing styles. Average assessment score performance is shown in each quadrant for different content areas, showing performance on the pre-test (before learning) and post-test (after learning). Signing styles for the lessons are represented by triangles with dashed lines (EM) and circles with solid lines (CM). The y-axis represents scores on the assessments, and the x-axis represents time (pre/post-test). Participants significantly improved their assessment scores from pre- to post-test across all conditions, with no consistent differences in learning based on signing style.

Our first hypotheses explored whether participants learned from our STEM video lessons, as measured by changes in their scores from pre-test to post-test. To do this, we added “time” (pre/post) as a fixed effect to our base model for pre- and post-test scores (*score ~ time* + *(1|topic)* + *(1* | *ID))*. We found a significant effect of time on test scores (*X*^2^(3) = 125.01, *p* < .001) while accounting for variability attributed to test topic and individual differences between participants. This model predicted a 22% improvement (1.76 points) from pre to post-test (*β* = 1.762, *t*(206) = −11.18). Indicating that participants learned from watching the video lessons and were able to significantly increase their scores on the post-test (Fig. 4).

For our second hypothesis, we wanted to investigate data from our matching task to further verify that we were measuring learning with the matching task by comparing it to the pre- and post-tests. To do this, we compared pre- and post-test scores, as fixed effects, to predict accuracy on the matching task as an outcome measure (*accuracy ~ pre-score* + *RT* + *(1| topic)* + *(1∣ID); accuracy ~ post-score* + *RT* + *(1| topic)* + *(1∣ID))*. We found that pre-test scores did not significantly predict matching task accuracy (*X*^2^(1) = 1.45, *p* = 0.228). However, post-test scores were significantly, positively related to increased scores on the matching task (*X*^2^(1) = 4.99, *p* = 0.026). Higher post-scores predicted higher accuracy on the matching task (*β* = 0.01, *t*(110.07) = 2.23). The fact that only post-test scores were correlated with the matching task provides further evidence that differences in the pre- and post-test scores were linked to learning. The remaining analyses for this paper will focus on the pre- and post-test scores. Results related to the matching task can be found in the supplementary information.

### Signing style

For our final research hypothesis, our aim was to investigate if differences in signing style were associated with learning. We predicted that content learned through CM signs would be associated with greater increases in scores from pre-test to post-test compared to the EM signing style. We tested this prediction this by adding signing style as an interaction effect to the baseline model: score ~ style * time + *(1|topic)* + *(1∣ID)*. We found that signing style did not have a significant main effect on test scores, *X*^2^(1) = 0.32, *p* = 0.570. The interaction between signing style and time was also not significant, *X*^2^(1) = 0.35, *p* = 0.556. Watching lessons in the different signing styles did not have an impact on increases between pre- and post-test score (Fig. 4).

However, we predicted that differences in learning from signing styles would be supported by differences in embodiment. As such, we also wanted to analyze how participants’ actual signed (and thus embodied) responses were related to their learning. Here, we considered participants’ own ASL summaries, looking at the individual signs produced after each lesson and the duration and accuracy of their explanations. The summary statistics for these variables can be found in Table 1. We compared these factors to their pre- and post-test scores. We separated the analyses based on the signing style of the lessons (i.e., analyzing the number of CM signs in the explanations after the CM lessons, and EM signs in the explanations after the EM lessons). The final models showed the effects of CM and EM sign use on test scores across both timepoints (i.e., *EM_score* ~ EM_use * time + *(1|topic)* + *(1∣ID)*. See supplementary information for analyses for all lessons combined.

**Table 1:** Summary statistics for participant signed summaries after each video lesson

	CM Count	EM Count	Summary Score	Duration
All lessons	M = 2.3 (SD = 3.6)	M = 5.1 (SD = 4.5)	M = 59% (SD = 37%)	M = 73 sec (SD = 28 sec)
Aligned Style	M = 4.5 (SD = 4)	M = 6.8 (SD = 4.5)	N/A	N/A

Aligned style includes only the CM signs produced in summaries signed after viewing CM video lessons and EM signs produced in summaries signed after viewing the EM video lessons.

For the EM lessons, there was no significant relationship between EM use and scores (*X*^2^(1) = 0.05, *p* = 0.830), nor was there an interaction effect between EM use and time (*X*^2^(1) = 1.32, *p* = 0.251). This analysis showed that there was no relation between participants’ use of EM signs in their own explanations of the STEM lessons and scores on the pre- or the post-test. We also looked at pre- and post-test scores as independent predictors, removing time as an interaction effect. We found that EM use was not significantly associated with scores at either time point (all *p*’s > 0.20).

**Fig. 5:**
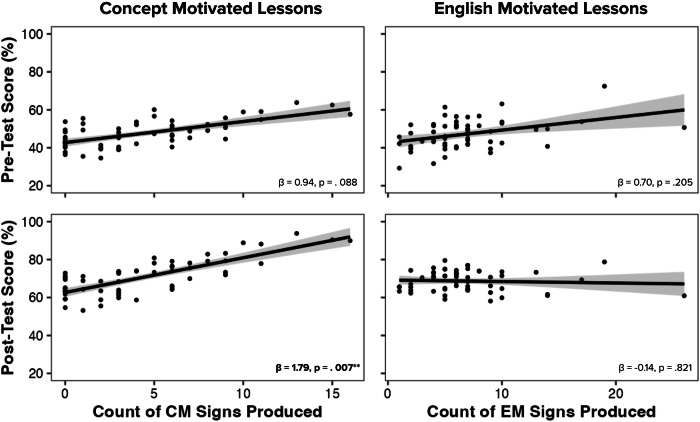
Relationship between types of signs produced and pre- and post-test scores. We compared the number of signs produced during participants’ explanations of what they had learned from each video lesson to assessment scores before the training phase (pre-test) and after (post-test). We compared the effects across signing style (CM vs. EM) and across time points (pre- vs. post-test). For the top two panels, the y-axes represent pre-test scores, and for the bottom two panels, y-axes represent post-test scores. For the two panels on the left, x-axes represent the count of CM signs produced after viewing CM lessons, and for the right two panels, the x-axes represent the number of EM signs produced after viewing EM lessons. Only the number of CM signs produced after CM lessons was significantly related to post-test scores, with each additional CM sign produced resulting in a 1.79% improvement in score. Each participant is represented by two points per signing style (two CM lessons and two EM lessons) for both the pre-test and the post-test for a total of eight points.

However, for CM lessons, there was a significant relationship between CM use and scores on both the pre- and post-test combined (*X*^2^(1) = 5.82, *p* = 0.016). More CM use was related to higher scores on both tests combined (*β* = 0.118, *t*(112.98) = 2.54). While the interaction effect for time and CM use was not significant, we were curious to see if the overall positive effect on test scores was driven by either time point. We investigated the differences in how CM use predicted pre- and post-scores independently (i.e., *CM_pre_score* ~ *CM_use* + *(1 topic)* + *(1∣ID); CM_post_score* ~ *CM_use* + *(1|topic)* + *(1∣ID))*. While CM use had a positive trend for predicting scores at both time points, only the post-scores are significantly associated with CM use (*X*^2^(1) = 7.24, *p* = 0.007; see Fig. 5). These results showed that using more EM signs had no relationship with test scores, but the use of more CM signs was related to higher scores, specifically higher post-test scores.

An additional factor that must be considered related to the analyses focused on sign use is the length of explanation. Participants who produced more CM/EM signs were likely to produce more signs overall and thus have longer explanations. It is possible that it was the length of the explanations, rather than the production of specific signs, that influenced test scores. Therefore, we also explored the effect of duration of their signed summaries on their pre- and post-scores. Similar to the sign use analyses, for lessons learned using EM signing, the length of participants’ summaries was not related to their pre- or post-test scores (all *p*’s > 0.40). Meanwhile, for the lessons learned using CM signing, the length of the signed summaries was not significantly related to their pre-scores (*X*^2^(1) = 2.05, *p* = 0.152). However, the length of their explanation was significantly related to their post scores (*X*^2^(1) = 11.02, *p* < 0.001). Longer summaries were related to higher post-test scores (*β* = 0.02, *t*(28.73) = 3.32). Spending more time summarizing a topic in the CM condition led to higher post scores, indicating more learning, while spending more time summarizing a topic in the EM condition did not.

Finally, we looked to see if a participant’s signed summary scores, which indicated the accuracy of their explanation during their summary, predicted their performance on the pre- and post-tests. Again, we found that for topics learned in the EM signing condition, there was no relationship between pre- or post-test scores and their summary scores (all *p*’s > 0.20). However, for topics learned in the CM signing condition, summary score was related to both pre-test (*X*^2^(1) = 5.03, *p* = 0.025; *β* = 0.99, *t*(45.71) = 2.25) and post-test scores (*X*^2^(1) = 18.03, *p* < 0.001; *β* = 2.10, *t*(54.15) = 4.25). For both pre- and post-tests, a higher explanation score was related to a higher test score.

Pre-test scores on CM topics were significantly related to scores for participants’ signed summaries. This result implies that prior knowledge was significantly related to how well participants would be able to explain topics after viewing the video lessons in the CM condition but not in the EM condition. Further, the summary score in the CM lessons was significantly related to post-test scores for CM lessons, and again, not for the EM lessons. This result suggests that the accuracy of the participants’ explanation after learning from the CM lesson was related to improved performance on the post-test. That summary scores for the participants explanations in CM lessons were related to both pre-and post-test scores seems to suggest that not only do people with prior knowledge seem to glean more for the CM videos, but that producing those summaries might also lead to better performance on a later test of knowledge.

## Discussion

Historically, DHH students, especially those who had delayed access to language, have lagged behind their hearing peers in STEM classes^4,5^. The lack of STEM learning resources in a primary and accessible language, ASL, has likely contributed to the persistence of this performance gap for DHH students^11^. Many groups around the US and the world are working to develop more STEM resources in signed languages. Our goal in this research was to explore if learning STEM content through ASL with an emphasis on connections to English or connections to concepts would be most beneficial for students. We predicted, based on existing research on embodied STEM learning, that participants would learn the most when they moved their bodies in ways that were aligned with the underlying concepts^30,38^. Thus, for Study 1, we developed video lessons in each signing style. We then used these videos in Study 2 to test if participant learning would be different for content learned from EM and CM video lessons, comparing learning within each participant.

Before investigating differences in learning based on EM or CM signing style, we needed to explore whether we had measured any overall learning from our signed STEM video lessons. We found that participants were able to learn across all four STEM topics. We were able to quantify their learning through improvement from pre- to post-test scores with an average improvement of 22%. Additionally, we found that post-test scores, but not pre-test scores, were significantly associated with accuracy on the matching task, indicating that the matching task was able to concurrently measure learning. These results together show that participants learned from the video lessons split across four distinct topics, even with limited training time.

After establishing that our participants learned from the video lessons, we looked for differences in learning from the two different signing styles. First, we added signing style as an interaction effect with time in our test-score model, testing changes in performance from each signing style from pre- to post-test. We did not find any significant differences in the change between participants’ pre- and post-test scores related to signing style. Participants’ improvements in test scores due to learning were approximately the same regardless of whether they watched the CM-style videos or the EM-style videos during the training. We expect that this similarity in learning across signing styles is likely because all our participants were adult, fluent users of ASL who had years of experience adapting to and learning from different signing styles. Additionally, as shown in Study 1, both the CM and EM videos were high quality, clear explanations. This finding is supported by the lack of difference in learning related to the different signing styles. The fact that there were no differences in learning related to signing style did not support our initial hypothesis that CM signs would be related to improved learning. However, we continued to investigate if there could be differences related to the embodiment of CM signs by exploring the more embodied part of the training: the participants’ signed summaries in ASL.

Past research on embodied STEM learning suggests that observing iconic gestures is a less embodied experience than engaging one’s own body to produce these gestures^30,32^. As such, we wanted to analyze participants’ explanations of what they had learned, exploring what types of signs participants produced after viewing the CM and EM videos. We found that the signing style participants used in their summaries was significantly related to their post-test scores. First, using more CM signs in their summaries was significantly linked to higher post-test scores, while the use of more EM signs was not. Additionally, producing longer summaries and more accurate summaries after the CM lessons was linked to higher scores, while length and accuracy of summaries after EM lessons had no relationship with their test scores. These results suggest that producing a summary that incorporates more CM signs is related to more conceptually accurate, embodied representations of the content, leading to higher post test scores^30,36,40^. This finding is particularly interesting given the within-participant design of this experiment. It shows that, within each individual participant, using the CM signs supported learning in ways that using the EM signs did not.

The fact that the accuracy of participants’ CM style summaries predicted their increased performance on a test they took prior to watching the lessons indicated that participants with more accurate summaries had a stronger foundation in the content. We suspect that this stronger initial performance reflected a foundation that made it easier to recognize the iconicity of the signs and thus incorporate them in their signed summaries^46^. This explanation was supported by the result showing that the relationship between signed summary scores and pre-test scores was only significant for the CM style lessons, not the EM style lessons. The CM style signs relied on more conceptual knowledge to understand the added visuospatial information conveyed in the signs while the EM style signs do not. Related results were found for iconic gesture use in hearing students^35^. For example, advanced students used more accurate and concrete gestures when explaining a calculus concept as compared to introductory students. In the case of our results, the CM signs are analogous to the more concrete gestures and have the potential for participants with prior knowledge to acquire and integrate these signs when summarizing what they learned.

Further evidence for this explanation comes from the structure of the videos in the CM condition. The CM-style videos intentionally did not pause during the lessons to explain why the CM signs looked the way they did. We avoided adding these explanations for two reasons. First, adding the explanations could have cued participants to differences between the styles of the videos they were watching. Second, providing additional elaborated explanations in the CM video lessons compared to the EM video lessons that would have created a bias towards learning from the CM condition. A potential alternative for future studies would be to include an explanation of the meaning of each part of the CM style signs, explaining why each sign was produced in a specific way that uses visuospatial information. Participants with a weaker foundational knowledge could especially benefit this more explicit description of the visuospatial information built into those signs. The results from this study provide support for the idea that embodied representations of STEM concepts created by using CM signs supported learning in ways that embodied representations of English words through EM signs did not.

Across these two studies, we successfully created STEM video lessons in two different signing styles and showed that participants could learn from those lessons. Our results demonstrated that there were no overall differences in learning after viewing the lessons through two signing styles. However, when participants were able to use and integrate concept-motivated (CM) signs into their summaries of what they had learned, it predicted higher post-test scores while the use of English-motivated (EM) signs did not. Specifically, in Study 1, we were able to create and validate pairs of videos on four different STEM concepts in two distinct, but equally clear, signing styles. One signing style emphasized the connections between ASL and English (EM) while the other emphasized the visuospatial connections to concepts more broadly (CM). The equity in the explanations between signing styles was further supported by results in Study 2. Participants scores improved from pre- to post-test after watching the ASL video lessons and learning equally from content presented in each signing style. However, when we investigated participant’s signed summaries, we found differences in how their CM style and EM style explanations were related to their pre- and post-test scores. For lessons that participants watched in the EM style, the EM signs produced while summarizing what they had learned had no relationship with their test scores. However, for lessons they watched in the CM condition, the more frequently they used the CM style target signs, and the longer and more accurately they summarized the topic, the higher the participants’ test scores, especially on the post-tests. These results suggest that specifically embodying the concepts through visuospatial, CM signs supported learning, particularly for participants with more prior knowledge, who could more easily understand the iconicity of those signs.

There are several important limitations in these studies that should be considered in the interpretation of our results. First, because Study 1 was an online study with different recruitment strategies than Study 2, our demographic characteristics were quite different between the two studies. In particular, Study 1, our sample included a majority of hearing participants, while our sample in Study 2 included a majority of DHH participants. While the videos might be clear and enjoyable for hearing participants, for most of whom English is their primary language, this might not be true for DHH participants who rely on ASL as a primary language. However, our results showing that participants in Study 2 learned equivalently from both signing styles provide support for the findings from Study 1 showing the equivalence of the lessons in each signing style. Additionally, our study focused only on adult participants with fluency in ASL and comfort with English. To better understand how CM signs could support DHH students, future research should be done with elementary and secondary students. Future work should also consider including participants with a wider range of language abilities.

Another important consideration for this study is the potential role of practice effects. Because our study was specifically focused on learning, we compared participants’ initial test scores to their performance on a similar test after training. However, it is possible that, regardless of the training, participants may have improved their scores through practice effects^47^. We did not incorporate a no-training control group to rule out the possibility of improvements in test scores coming from test-retest effects, which means the gains we saw from pre-test to post-test could be attributed to learning from taking the test twice in the same day instead of from the videos themselves. We chose not to include a control group because our primary focus in this study was to compare within-participant learning to fully utilize participants’ time in the lab. Further, our pre- and post-tests were structured to try to reduce some of these practice effects by randomizing the presentation of the questions and including alternative versions of questions (e.g., one version of the question asks about an acid, the alternative asks about a base). We also used additional measures of learning (i.e. the matching task and participant’s signed summaries) that were not repeated before and after the lessons and which aligned with the learning we saw from the pre- and post-tests. Future research could explore the comparison to a group of control participants who take the pre- and post-tests without being exposed to any of the training to rule out overall improvement due to practice effects.

Additionally, it is possible that such a brief training with a complex STEM topic was not sufficient to observe true differences in learning or signing style, especially because we intentionally chose to not explain the motivations behind the CM signs. For example, if participants had the chance to spend more than four to six minutes learning for each topic, or if there were several days between training and assessments, the effects of learning across time or the influence of embodiment through signing styles might become more apparent. For example, one study that looked at embodied learning through virtual reality found that there was no difference between embodied and non-embodied conditions in an immediate post-test. But two weeks later, participants in the embodied condition had retained more than the control group^48^. Future studies should consider including explanations of the CM sign motivations and additional repeated, delayed post-test measures to observe how extended time and consolidation influence the learning process. Future research could also consider adding a more hands-on learning component within virtual reality or the use of manipulatives to increase embodiment and additional measures outside of assessment scores, like measures of engagement with the topics^49^. These measurements, which may include more sensitive measures of emotional experience or engagement may pick up on differences in feelings, attention, or cognition that may be key for learning.

Overall, our results suggested that using concept-motivated signs supported embodied STEM learning in signing learners. These results should inform the development of future STEM signs and ASL STEM resources that emphasize conceptual and visuospatial connections between signed language and underlying STEM concepts. This study also provides important insight into how to best use those resources with DHH students. The results suggest that CM signs support learning when they are embodied by learners, especially when they incorporate signs into their own explanations of what they have learned. Simply watching CM-style video lessons in ASL might not be sufficient to glean the benefits of embodied cognition through visuospatial language. Thus, educational professionals should encourage students to use the signs they are learning about through copying, discussion, and summarizing to create more embodied representations of the concepts. Future directions of this research would be well-positioned to examine how embodying these signs may be associated with augmented learning. Studies testing the impact of utilizing these signs in real-world educational environments would provide more direct insight into how best to encourage students to engage with these signs by practicing and incorporating them into their STEM ASL vocabulary. The ultimate goal of STEM education is to help students develop foundational, conceptually accurate representations of information that they can continuously build upon throughout their education. The results of this study suggest that learning through ASL, especially when signs provide visuospatial and concept-motivated information, can help students build embodied representations of STEM topics.

## Methods

### Study 1 topic selection

We first selected the picking topics for the four video lessons. We used the following criteria to identify potential topic areas:

1. The topic would likely be taught in upper-high school and early college-level courses.
2. The topic already had existing CM and EM signs available in a widely accessible online ASL STEM lexicon database.
3. The key concepts could be explained clearly in a four to six-minute video lecture.
4. Topics came from a variety of STEM subfields.

After applying these criteria, we chose the following topics: (1) Brønsted–Lowry acids and bases (chemistry), (2) graphing first derivatives (calculus), (3) normal and skewed distributions (statistics), and (4) stress and strain curves (engineering).

### Study 1 script and video development

After identifying our four topic areas, we developed videos for each, using an iterative method that incorporated feedback from research team members who were fluent in ASL, as well as experts in each field. First, we developed outlines in written English for each topic. Both styles represented fluent signing in ASL but differ in strategies used to present key concepts and vocabulary. The overall goal was for the CM videos to maximize the use of the spatial nature of ASL and incorporate the CM signs identified from ASL STEM lexicon databases. Meanwhile, the EM translations used a version of ASL that aligned more closely with the linear style of English and used more fingerspelling and non-technical signs. Because both videos for each topic were based on the same outline and core content, differences between them were created by the use of CM and EM signs for specific keywords and overall differences in signing style, not by the information conveyed in each version of the video.

Each pair of videos for each topic area went through three to five rounds of internal review for accuracy of the content and appropriateness for upper-high school level classes. Videos were also reviewed to create a balance between consistency and distinctness between signing styles. In order to provide feedback on preliminary versions of each video, the team used the online video assessment software, GoReact^50^, which allowed the draft videos to be annotated with video feedback in ASL. Our review team was made up of seven scientists with backgrounds in deaf education, STEM sign development, and educational neuroscience. All members of the internal review team were fluent in ASL; three were deaf scientists, and a fourth was a child of deaf adults whose first language is ASL.

After our internal review team approved the videos, we identified one to three deaf signing experts for each topic area to evaluate the videos. Content-experts had a graduate-level degree related to the topic area and experience teaching about the topic using ASL. We requested feedback from fluent ASL users with expert knowledge about the selected topic who were naïve to our goal to differentiate between EM and CM styles. Content experts were asked to assess each video individually and then compare them. Experts were asked to provide feedback about the scientifically accuracy of the content, if they had concerns about any of the images used, if they had any comments on specific signs, and if they found the signing to be generally comprehensible and likely to be seen in a high school or college classroom. Three pairs of videos (acids and bases, normal distributions, and graphing derivatives) required only minor adjustments for accuracy and consistency. The initial video lesson on stress-strain curves was deemed too complicated for an introductory course, so the content was simplified. The second video draft went through the internal and external review process a second time. Through this iterative process, we developed final signed versions of each video lesson. These videos were confirmed to be accurate and appropriate by the research team and external experts in the topic. After all video pairs were verified for accuracy by our content experts, we moved to validating the videos with key stakeholders: professionals working in deaf education with varying levels of knowledge about our chosen STEM topics.

### Study 1 participants

Participants were recruited to join in an online study evaluating the signed video lessons. Participants were screened to be between the ages of 18 and 60, able to read and write in basic English, and have self-reported advanced fluency in ASL. All participants confirmed that they worked in the deaf community as a teacher of DHH students or an ASL interpreter, ASL linguist, or ASL teacher in the United States. Participants were recruited through flyers, emails, social media posts, and word of mouth. As a result of posting on social media, the survey was overwhelmed by “bots” who provided false responses, resulting in *N* = 110 total responses. We used the following criteria to screen for genuine responses. First, we eliminated anyone who spent less than 5 minutes on the survey as this was insufficient time for them to view the two videos. Second, we eliminated anyone who had the same answer for all of the multiple-choice questions. These participants were excluded since they should not have the same answer for questions asking about both positive and negative qualities (i.e., choosing the same video as the most enjoyable and the most frustrating). We also eliminated participants who provided multiple single-word answers or “no comment”/”na”. There were only three open-response questions, so entirely non-substantive answers were considered criteria for exclusion. Finally, we eliminated anyone who answered the signing style questions with nothing about the signing style (i.e., “this is to benefit deaf and dumb students”, “I got a bit confused”, “stress this is how stress affects one”). Using these criteria, we reduced our included responses to N = 30.

Participants (*N* = 30) self-reported gender: 27% “Male” (*n* = 8), 63% “Female” (*n* = 19), 10% “Nonbinary” (*n* = 3). Participants self-reported race (participants could select more than one option): 73.3% White, 23.3% Black or African American, 3.3% American Indian or Alaskan Native, 3.3% Asian, and 3.3% chose to self-describe. Most participants did not identify as Latino/a/x (90%), and 10% identified as Latino/a/x. Participants self-reported age: 30% (*n* = 9) aged 18–30, 40% (*n* = 12) aged 31–40, 16.7% (*n* = 5) aged 41–50, and 13.3% (*n* = 4) aged 51–60. A majority of participants described themselves as hearing (67.7%), 17.7% chose D/deaf, 13.3% chose hard of hearing, and 3.3% chose to self-describe. See Table 2 for all participant summary statistics. All parts of this study involving human subjects were approved by the Gallaudet Institutional Review Board. All participants provided informed consent. Any images or videos used in this manuscript were from participants who also provided written consent to share their images without anonymity. IRB approval reference number: IRB-FY23-132. Participants were given an online gift card as compensation for their voluntary participation.

**Table 2:** Summary statistics of demographics for online respondents from Study 1

*Survey*	*%*	*Description*
Age		“What is your current age?”
18–30	30	
31–40	40	
41–50	16.7	
51–60	13.3	
Gender		“How do you describe yourself?”
Female	63	
Male	27	
Non-binary	10	
Race		“Choose one or more races that you consider yourself to be:”
White	73.3	
Black/African American	23.3	
American Indian/Alaskan Native	3.3	
Asian	3.3	
Self-describe	3.3	
Prefer not to share	0	
Latino/a/x or Hispanic		“How do you describe yourself?”
Yes	10	
No	90	
Hearing Status		“Please choose the option that best describes your hearing status:”
D/deaf	17.7	
Hard of Hearing	13.3	
Hearing	67.7	

A majority of participants from Study 1 were white, non-Latine, hearing females, between the ages of 18 and 40.

### Study 1 external video validation procedure

In order to validate the video pairs, we ran an online study implemented in Qualtrics software^51^ to survey key stakeholders in deaf education and experts in ASL: STEM teachers, ASL-English interpreters, ASL linguists, and ASL teachers. We chose these groups because of their expertise in deaf education and because they were part of a target audience who could potentially use the videos in a classroom setting. Our goal was to verify that we had not biased the content of the videos towards one style of signing or the other, for example, that one style of signing was not clearer overall. We also sought to investigate if reviewers, who were naïve to the intentional signing styles used to create the videos, would accurately describe the differences in the CM and EM signing styles.

Each participant first completed an eligibility screener followed by an online consent form. All participants provided written informed consent. The consent form was in written English with an ASL video translation available. After completing the informed consent, each participant was pseudo-randomly assigned a topic area and viewed a pair of videos that included both CM and EM signing styles. Participants were assigned to review a pair of videos in only one topic area. Videos were labeled as A/B and pseudorandomized for which version had which label to avoid priming reviewers to our goals. Participants were asked to describe the signing style in each video after viewing. Participants identified which video they found (1) more clear, (2) more enjoyable, and (3) more frustrating. For all multiple-choice questions, reviewers could choose video A, video B, both videos, or neither video. The survey took approximately 10-20 minutes to complete.

### Study 2 participants

Participants were recruited to participate in an in-person study. Participants were screened to be between the ages of 18 and 45, able to read and write in basic English, and have self-reported advanced fluency in ASL. Participants were recruited through flyers, emails, and personal connections in the Washington, D.C. metro area. Overall, *N* = 31 participants completed the screener survey and arrived at the lab for the in-person study. One participant withdrew from the study after completing the background information surveys and thus was not included in the rest of the study, leaving *N* = 30 participants who completed all measures in the study. All participants provided informed consent to participate in the study. All parts of this study involving human subjects were approved by the Gallaudet Institutional Review Board. All participants provided informed consent. IRB approval reference number: IRB-FY23-132. Participants were thanked for their time and compensated for their voluntary participation with an online gift card or cash payment.

Participants were between the ages of 20 and 44 (*M* = 29.2, SD = 7.52). Participants’ self-reported gender: 43% “Male” (*N* = 13), 47% “Female” (*N* = 14), 10% “Nonbinary” (*N* = 3). Participants self-reported race (participants could select more than one option): 56.7% White, 20% Black or African American, 3.3% American Indian or Alaskan Native, 6.7% Asian, 10% chose to self-describe, and 3.3% chose not to share this information. The majority of participants did not identify as Latino/a/x (80%), while 20% identified as Latino/a/x.

This group of participants represented a diverse community of various language backgrounds and self-reported hearing statuses. Participants were asked to “choose the option that best describes your hearing status.” A majority of participants described themselves as D/deaf (73.3%), 13.3% chose hard of hearing, and 13.3% chose hearing. Although participants in this group of DHH and hearing participants reported having advanced fluency in ASL, their experience with and age of acquisition for ASL varied (see ASL-age, Table 3). There is slightly less variation with the age at which participants started to learn English (spoken or written), with the vast majority of participants learning English before age 4 (see English-age, Table 3).

**Table 3:** Summary statistics for demographics and language experiences for in-person participants in Study 2

Survey	Mean (SD)	Scale Range (Observed)	Description
Age	29.2 (7.52)	18–45 (20–44)	“What is your current age?”
Gender	%	N/A	“How do you describe yourself?”
Female	43		
Male	47		
Non-binary	10		
Race	%	N/A	“Choose one or more races that you consider yourself to be:”
White	56.7		
Black/African American	20		
American Indian/Alaskan Native	3.3		
Asian	6.7		
Self-describe	10		
Prefer not to share	3.3		
Latino/a/x or Hispanic	%	N/A	“How do you describe yourself?”
Yes	20		
No	80		
Hearing Status	%	N/A	“Please choose the option that best describes your hearing status:”
D/deaf	73.3		
Hard of Hearing	13.3		
Hearing	13.3		
Language Preference			Asked about communication preferences; higher scores = stronger preference
ASL	4.53 (1.03)	−5 to +5(0 to +5)	
Signed Exact English	-1.48 (3.04)	−5 to +5 (−5 to +4)	
Written English	3.66 (1.95)	−5 to +5 (−2 to +5)	
ASL Comfort	3.66 (1.95)	1–5 Disagree–Agree (4–5)	Asked about comfort with ASL expressive and receptive skills and role in identity, higher scores = more comfort with ASL
Years ASL	20.5 (10.4)	(2–42)	"How many years have you been using ASL?"
ASL-age	**%**	N/A	"What age did you start learning ASL?"
0–2 yrs	40		
2–4 yrs	10		
4–10 yrs	13		
10–13 yrs	13		
14–17 yrs	7		
18–22 yrs	13		
23–26 yrs	3		
26 + yrs	0		
English-age	**%**	N/A	"What age did you start learning English?:
0–2 yrs	46.7		
2 –4 yrs	36.7		
4 –10 yrs	3.3		
10–13 yrs	0		
14–17 yrs	0		
18–22 yrs	0		
23–26 yrs	0		
26 + yrs	0		

Participants in Study 2 were more likely to identify as d/Deaf than participants in Study 1. Summary statistics highlight the wide range of preferences and experiences for ASL, signed systems like Signed Exact English (SEE), and English.

### Study 2 pre- and post-tests

After developing our lectures, we developed pre- and post- tests focused on the content covered for each STEM topic. These tests allowed us to track how much participants were able to learn from the video lessons. For each topic, we developed eight questions with different answer types (e.g., multiple choice, multi-select, ranking, true-false). For each topic, at least two questions were designed to address each of the first three levels of Bloom’s taxonomy (remember, understand, apply^52^). Designing questions to test these different assessment levels made it more likely that we would observe variability on test performance on both the pre- and post-tests. All eight questions were roughly the same from pre- to post-test. We made slight modifications to decrease the chance that improvement would be from familiarity with the questions^47^. For example, in the pre-test, we asked which of four pictures was a base, while in the post-test, we asked which of four pictures was an acid.

At the start of the study, participants were randomly assigned to view two topic areas using CM signs and two topic areas using EM signs, and the signing style was counterbalanced between participants (i.e., approximately half of the participants saw material for acids and bases using CM signs, half saw EM signs). Participants first completed a pre-test assessment. An ASL translation of each question was provided at the top of each test page. Participants saw the same topics using consistent CM or EM signing styles for all pre- and post-tests, and video lessons, depending on which signing style would be used in the video lesson from which they would learn. For example, if a participant was assigned the CM style for the acid and base lesson, all eight questions related to acids and bases showed a CM style ASL translation for both pre- and post-test. Then, if they were assigned the EM style for the normal distributions lesson, all eight questions for normal distributions would have the EM style translation visible for both tests. Questions were presented in four blocks organized by STEM topic. The order of blocks and the order of questions within each block were randomized. Because the answer choices were signed in the translation videos, these were not presented in a randomized order. Questions in which answers used images were randomized.

### Study 2 matching task

**Fig. 6:**
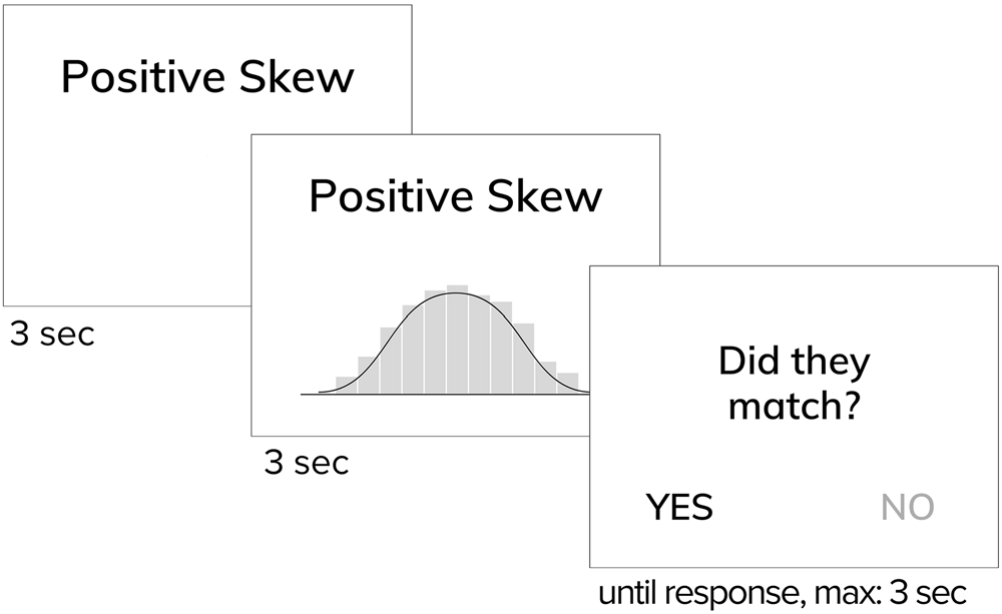
Diagram of matching task. Diagram showing logic and the timing for the cognitive matching task. Participants completed 204 trials, half of which had matching words and images and half of which were mismatched.

In addition to the pre- and post-tests, we developed a matching task to measure learning by exploring whether participants could match words and images drawn from each STEM topic. This task allowed us to assess accuracy under time pressure while participants decided whether the word and the picture matched the same concept. To create the word and picture stimuli, we identified four to five key terms or phrases for each lecture and created six images related to each term (17 words, 102 images). We used PsychoPy software^53^ to present each English word or phrase for 3 s; the word would remain on the screen with an image for three additional seconds (six total seconds). The word and image would then disappear and be replaced with a screen asking, “Did they match?” The location of the responses (yes/no) was randomly assigned within each trial to prevent participants from anticipating a response. See Fig. 6 for an example trial. Participants were presented with each word/phrase 12 times, each time paired with a different image, for a total of 204 trials. The word and the image matched for 50% of trials. For the mismatched trials, half of the images were from the same topic as the word (e.g., word: brittle, image: elastic), while the other half were from a different topic than the word (e.g., word: brittle, image: skew).

### Study 2 measures of academic experiences

Participants responded to a variety of different questions about themselves, including demographic information and questionnaires about academic anxieties, experiences, and performance. Questionnaires were presented in a randomized order for each participant. Most questionnaires included optional short videos of the instructions and questions in ASL. All videos were signed by a fluent deaf signer in a neutral signing style (e.g., not CM or EM). Summary statistics for these surveys can be found in Table 3. Measures of academic anxiety and spatial habits were collected but not used for analyses included in this paper and will be reported in an additional manuscript.

### Study 2 procedure

For Study 2, participants completed the pre-test, the training portion by viewing the video lessons developed in Study 1, and the matching task and post-test to measure their learning (see Fig. 3 for an overview of the procedures). First, participants completed an online version of the informed consent, background surveys, and questionnaires. Participants were given the option to complete these online tasks within 24 h before arriving at the lab or as the first part of the in-lab study. These online tasks took ~30–45 min to complete. Next, participants completed the multiple-choice pre-test on the four STEM topics included in the training. The questions were presented in blocks, so all eight questions related to each topic area were presented together, in a randomized order. Questions focused on identifying and using the main concepts, terms, and figures featured throughout the video lessons. The pre-test took 10-20 minutes to complete.

**Fig. 7:**
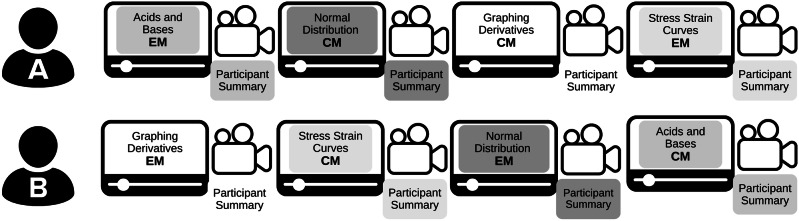
Two example training sequences. Graphical summary showing two examples of the six counterbalanced video lesson sequences.

After completing the pre-test, participants engaged in the learning/training portion of the study. Participants watched four video lessons depicting all four STEM topics: acids and bases, graphing derivatives, stress strain curves, and normal distributions. Video lessons were presented in one of six pseudo-randomized orders (2 example orders in Fig. 7). Each participant saw all four STEM topics, counterbalanced to have two in the visuospatial, CM version of the video and two in the more linear, EM version of the video. Participants always watched all four topics and always learned two topics in the CM style and two topics in the EM style of signing. Importantly, the signing styles were counterbalanced across topics between participants (i.e., “acids and bases” presented in both the CM and EM condition, depending on the sequence assigned). After watching each video lesson, participants were prompted to summarize what they had learned in 1–2 min, signing their response in ASL. Their signed summaries in ASL were recorded with a webcam. Due to a software error, videos of one summary for three different participants were not recorded. The training took 25–35 min, depending on the length of the participant summaries.

After the training, participants completed the matching task described above (Fig. 6). The matching task started with 7 practice trials so participants could become accustomed to the task, particularly the swapping of the responses from the left and right sides. Participants were prompted to take a break every 34 trials. The matching task took 35–45 min to complete. Electrodermal activity was collected during the training portion and the matching task. This data was not used in the analyses included in this paper and will be explored in other manuscripts. After completion of the matching task, participants completed the post-test.

### Study 2 pre- and post-test and signed summary scoring

For pre- and post-test scoring, participants earned credit for correct answers and received no credit for incorrect answers. For questions with multiple possible correct responses, answers were weighted based on how many correct answers possible for each question. For example, if a question only had one correct answer, it was given a weight of 1. However, if a question had three possible correct answers, each was assigned a weight of 0.33. The maximum possible score for each of the four topic tests was 8 points, for a total maximum test score of 32 points.

We scored the participants’ signed summaries using two methods. First, we counted how many times each participant produced specific key words from each video lesson. Their signs were categorized as either matching the CM, EM, or other version of the sign. A reference file with brief videos (GIFs) of the target signs was used while coding the participant videos. Slight deviations from the target signs were acceptable for the CM signs as long as they were still accurately representing the concept. For fingerspelled EM signs, slight deviations in spelling were also acceptable as long as they were identifiable as the target word. We then counted all of the uses of CM and EM signs for each participant for each topic. For the analyses in this paper, we focused on the signs that were produced when the signing styles matched. Specifically, we counted the number of CM signs that were produced after viewing CM video lessons and the number of EM signs produced after viewing the EM lessons.

In addition to counting the use of different signs, we also scored participants for how accurately they explained key concepts from each video lesson. For each video lesson, we identified three to five possible concepts from the lesson participants should explain. They were given credit for any concept they accurately described in their summaries. Summaries were scored as a percentage of accurate topics included in the summary, with a maximum possible score of 100% for each topic. Simply naming a concept was not sufficient to earn credit; some accurate expansion on the topic had to be expressed to be given credit. See Table 1 for summary statistics from participants’ signed summaries.

Videos were coded using the software ELAN^54^. The primary coder was a deaf, fluent signer. After coding of all videos was completed, two secondary coders, proficient in ASL, reviewed the ELAN scripts for accuracy and made corrections for any errors. We utilized R^44^ the *irr* package^55^ to calculate consistency between raters and used. We computed intraclass correlation coefficients for CM count, EM count, and summary scores for each topic. All scores were above 84%, indicating good to excellent correlation between raters. The sign counts and summary scores following the second review were used for the analyses in this paper.

## Supplementary information

Supplementary Information

## Data Availability

Due to the small nature of the DDBHH communities, the researchers are not able to publicly share all behavioral data collected to protect participant confidentiality and privacy. Supplementary information, an appendix of materials, and preprint versions of this manuscript are available through the Open Science Framework through PsyArXiv: osf.io/djwqe. Researchers should contact the corresponding author to request access to raw data or data that includes demographic information. We report how we determined our sample size, all data exclusions (if any), all manipulations, and all measures in the study. This study’s design and its analysis were not pre-registered.

## Code availability

The R code used for analysis will also be available through the Open Science Framework osf.io/djwqe. The code was written using R 4.4.1 (2024-06-14).
