# Supplementary material for: Producing concept-motivated signs supports learning of STEM in American Sign Language
Source: NPJ Sci Learn. 2026 Apr 11;11:37. doi: 10.1038/s41539-026-00418-6 (PMC13254344; doi:10.1038/s41539-026-00418-6)
Supplement: Supplementary file 1 — Supplementary Information [file 41539_2026_418_MOESM1_ESM.pdf]

Supplementary Materials

Study 1: Results

Supplementary Figure 1.

Bar graph showing reviewer preferences broken down by topic area

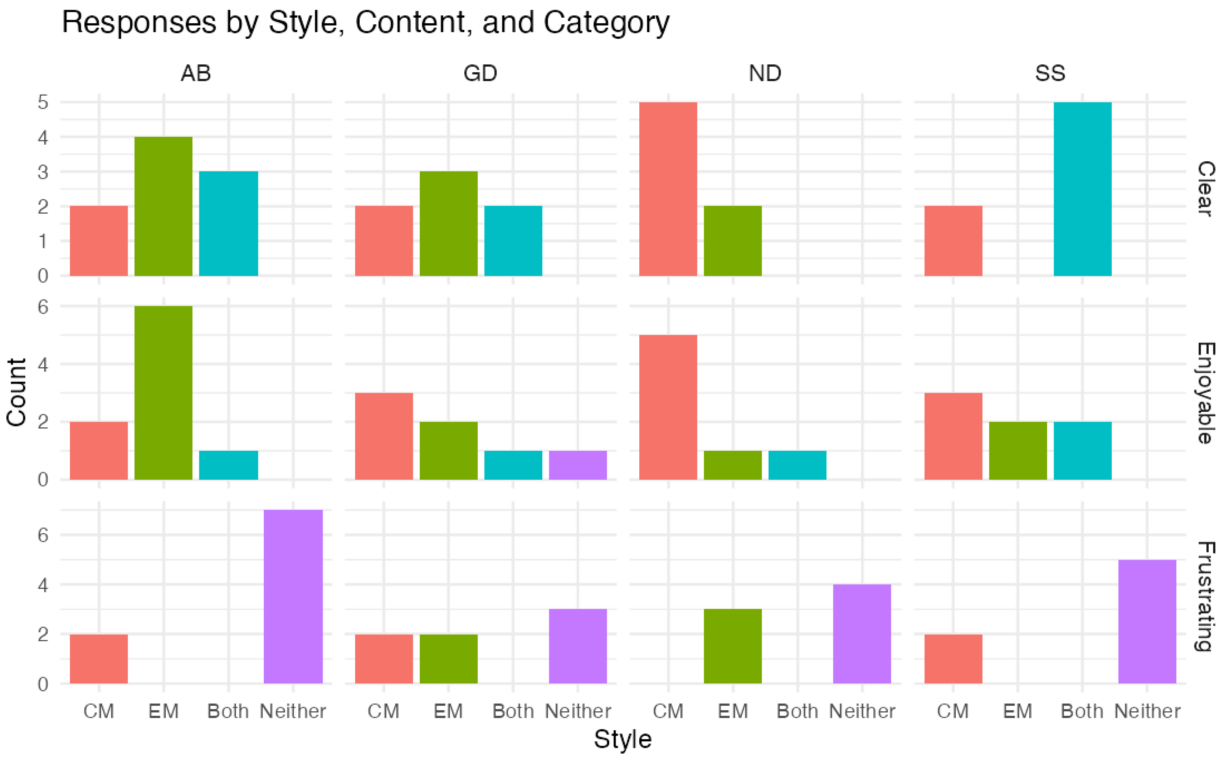

Note. Acids and Bases (AB, n = 9); Graphing Derivatives (GD, n = 7); Normal Distributions (ND; n = 7); Stress-Strain Curves (SS, n = 7). Columns for each individual graph are in the order: concept-motivated, English-motivated, both, neither, for each video. The rows are for which video they found most clear, most enjoyable, and most frustrating. There are no conditions under which all participants chose only one style of video (i.e., all responses for most clear are for concept-motivated and none are for, English-motivated, both, and neither).

Study 2: Results

Signed Summaries for All Lessons Predicting Assessment Scores

We looked at how signed summaries predict assessment scores when looked across all lessons, not just those that matched the signing style being produced. For these analyses, we used models following this pattern: *score ~ EM_count * time + (1|ID)*. For EM use, there was no significant relationship between the use of EM signs and scores ($X^2(1) = 2.22$, $p = .136$), nor were there any interaction effects between time and EM sign count ($X^2(1) = 0.238$ $p = .626$). However, use of CM signs did significantly predict assessment scores ($X^2(1) = 4.82$, $p = .028$) with a higher count of CM signs relating to high scores ($\beta = 0.07$, $t(207.03) = 2.26$). But the interaction between CM signs and time was

not significant ($X^2(1) = 0.89$, $p = .346$). Neither EM use nor CM use predicted accuracy on the cognitive task (all p's > .60).

Signed Summaries Predicting Accuracy for the Matching Task

We also looked at how the different variables for the signed summaries predicted accuracy on the matching task, following this pattern: *accuracy ~ variable + RT + (1| topic) + (1| ID)* (**Supplementary Table 2**). We found that only the score for their explanations after viewing videos in the concept-motivated condition predicted accuracy on the matching task. The number of times they used CM and EM signs, the length of their explanations, and the score for their explanation after viewing EM videos were not significant predictors of their performance on the matching task.

Supplementary Table 1.
Results for signed summary variables predicting accuracy on the matching task.

Variable of interest	Results
EM_count	$X^2(1) = 2.2911$, p = .1301
CM_count	$X^2(1) = .5627$, p = .453
Duration_EM	$X^2(1) = 2.2912$, p = .1301
Duration_CM	$X^2(1) = 2.7795$, p = .0955
Explain_score_EM	$X^2(1) = 0.2637$, p = .608
Explain_score_CM	$X^2(1) = 2.8561$, **p = 0.0496**<br>$\beta = 0.0606$, t(48.797) = 1.964

Videos of ASL Signs Described in Manuscript
Videos of example signs can be found in the online file repository: osf.io/djwqe
